# Supplementary material for: Integrated collagen architecture and composition improve risk stratification in triple-negative breast cancer
Source: Neoplasia. 2026 Sep 12;81:101367. doi: 10.1016/j.neo.2026.101367 (PMC13587055; doi:10.1016/j.neo.2026.101367)
Supplement: Supplementary file 1 [file mmc1.pdf]

Supplementary Figure 1

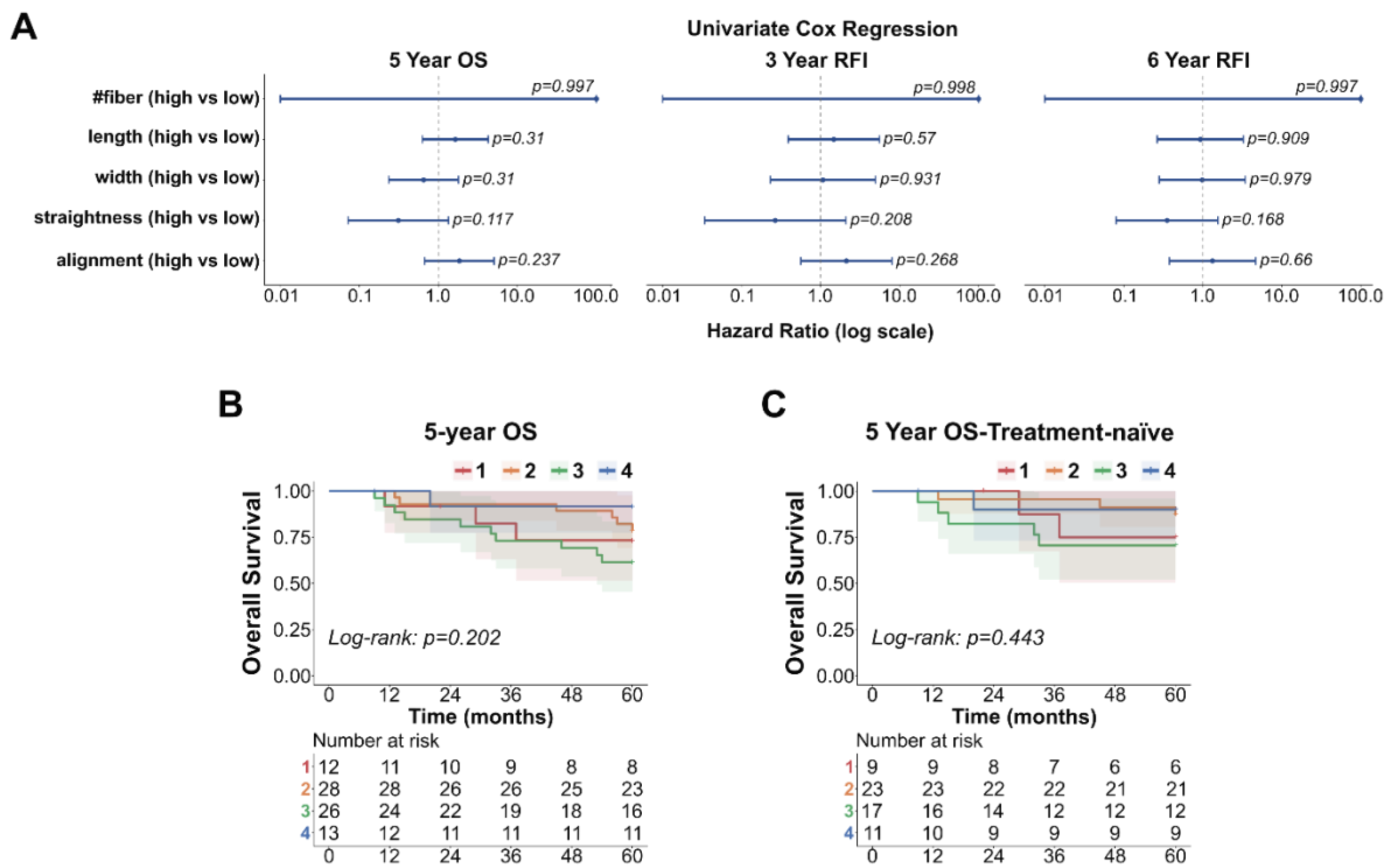

Supplementary Figure 1. Quantitative collagen phenotyping from Masson's Trichrome histology. A, Forest plots of univariable Cox proportional hazards regression for individual collagen features (fiber count, length, width, straightness, and alignment) across 5-year OS, 3-year RFI, and 6-year RFI. **B–C**, Kaplan–Meier curves for 5-year overall survival across all four collagen clusters in the full cohort (B; log-rank p=0.202) and in treatment-naïve patients (C; log-rank p=0.443). Shaded areas represent 95% confidence intervals.

Supplementary Figure 2

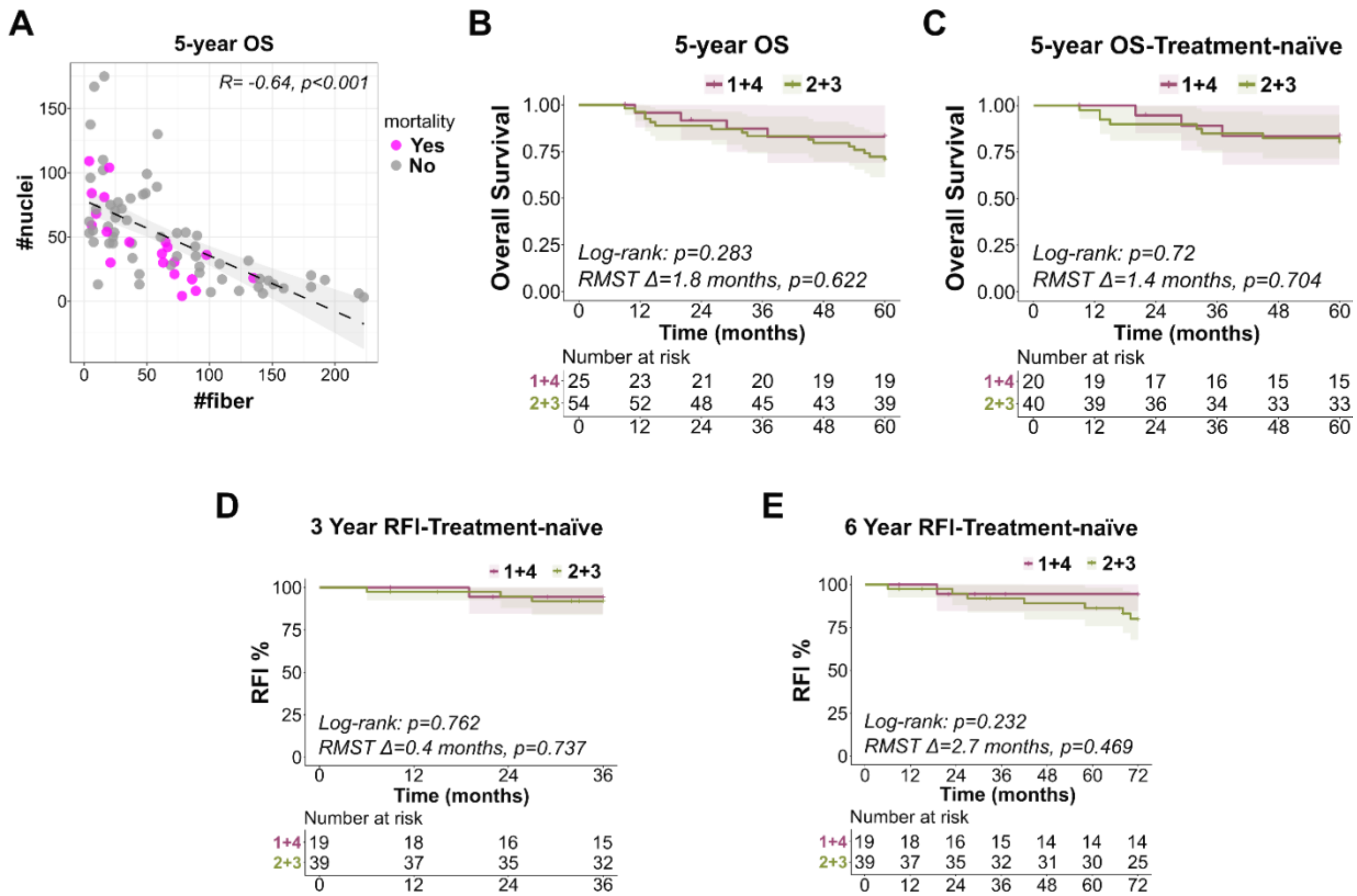

Supplementary Figure 2. Collagen cluster survival analysis. A, Scatter plot showing the correlation between fiber count and nuclei count, overlaid with 5-year mortality status (Spearman R=−0.64, p<0.001). **B–C**, Kaplan–Meier curves for 5-year overall survival comparing Clusters 1+4 and Clusters 2+3 in the full cohort (B; log-rank p=0.283; RMST Δ=1.8 months) and in treatment-naïve patients (C; log-rank p=0.72; RMST Δ=1.4 months). **D–E**, Kaplan–Meier curves for recurrence-free interval (RFI) in treatment-naïve patients comparing Clusters 1+4 and Clusters 2+3 at 3 years (D; log-rank p=0.762; RMST Δ=0.4 months) and 6 years (E; log-rank p=0.232; RMST Δ=2.7 months). Shaded areas represent 95% confidence intervals.
